# A mixing–isolation–mixing model of speciation can potentially explain hotspots of species diversity

**DOI:** 10.1093/nsr/nwy112

**Published:** 2018-10-08

**Authors:** Richard J Abbott

**Affiliations:** School of Biology, University of St Andrews, UK Reviewer of NSR

From comparative genomic analyses between species and population genetic analyses within species, He *et al.* [1] provide convincing evidence that mangrove divergence across the Malacca Straight, separating the Indian and Pacific Oceans at the Malay Peninsula, Southeast Asia, fits a model of speciation occurring over a time span that includes cycles of isolation (allopatry) interspersed by periods of mixing (gene flow). Key to this demonstration was the known biogeography of the region, and the fact that rises and falls in sea level during the period of divergence and speciation have repeatedly opened and closed the Malacca Straight to migration between the Indian and Pacific Oceans on either side of the Strait. Without this knowledge, it would have simply been concluded that speciation had occurred with gene flow, thus failing to recognize the occurrence of allopatric phases and their likely importance in the build-up of reproductive isolating incompatibilities between taxa [2].

More generally, He *et al.* [1] show that their proposed mixing–isolation–mixing (MIM) model of speciation can result in a large increase in the number of species that form in areas where impermanent geographical barriers allow recurrent cycles of isolation and mixing to occur. This is the case if speciation occurs after each of several isolation phases, which may explain why hotspots of species diversity occur in some regions where such impermanent geographical barriers (rather than longstanding ones) are present. They further suggest that the MIM mechanism of speciation may explain the occurrence of rapid species radiations in some taxonomic groups. Possible examples include the radiation of cichlid fish in Lake Victoria, East Africa, where repeated cycles in the rise and fall of water levels have occurred during the period that covers this radiation, and the radiation of the plant genus *Nigella* across the Aegean Archipelago in the Mediterranean [3]. The Aegean Archipelago originated ∼5 Ma, with some islands repeatedly connected and disconnected during the Pleistocene due to falls and rises of sea levels in glacial and interglacial periods, respectively. The terrestrial flora and fauna of this and other continental shelf island archipelagos provide ideal material for further testing of the MIM mechanism.

The MIM mechanism of speciation is of further relevance to an understanding of hybrid zone formation between taxa that are not fully reproductively isolated from each other. Formation of hybrid zones may occur in either of two ways, by primary intergradation where divergence occurs in the face of gene flow or by secondary contact between populations that diverged in allopatry [4,5]. However, hybrid zones formed in either way exhibit very similar patterns of genetic and phenotypic variation at equilibrium [4,6,7], and, consequently, their exact mechanism of origin cannot be determined in the absence of a historical record. Attempts have been made to reconstruct from genomic data the way in which some hybrid zones have originated, leading to the conclusion that an origin in the face of continuous gene flow (primary intergradation) is a satisfactory explanation in some instances (see, for example, Filatov *et al.* [8]). However, without a historical record, it is not possible to rule out the occurrence of allopatric phases, which may have been key to the development of prezygotic and postzygotic isolating barriers that reduce gene flow between parental types [9]. The convincing demonstration of the MIM model to explain mangrove speciation across the Indo-Pacific Barrier at the Malay Peninsula sets the scene for future studies of speciation (and the origin of hybrid zones) in all regions where an underlying historical record of biogeography is available or can be obtained.
